# Subtypes of digital literacy and their association with innovative behavior among Chinese undergraduate nursing students: a latent profile analysis

**DOI:** 10.3389/fpubh.2026.1717234

**Published:** 2026-02-06

**Authors:** Xueyan Wang, Yingying Wang, Xinyue Chen, Siyuan Lv, Wanyu Ding, Shaoyong Ma, Mingfen Tao

**Affiliations:** 1 Graduate School, Wannan Medical College, Wuhu, China; 2 Emergency Intensive Care Unit, Yijishan Hospital of Wannan Medical College, Wuhu, China; 3 School of Nursing, Wannan Medical College, Wuhu, China; 4 Department of Nursing, Yijishan Hospital of Wannan Medical College, Wuhu, China

**Keywords:** digital literacy, innovative behavior, latent profile analysis, nursing, student

## Abstract

**Introduction:**

Digital literacy has become a core competency for nursing professionals, enabling them to adapt to modern healthcare environments and engage effectively with emerging technologies. It is closely linked to innovative behavior, which is essential for problem solving and advancing nursing practice. Despite its importance, limited research has examined differences in digital literacy among undergraduate nursing students and how these differences influence innovation.

**Methods:**

A cross-sectional study was conducted using a convenience sample of 450 undergraduate nursing students from four universities in Anhui Province, China. Participants completed a general information questionnaire, the Undergraduate Digital Literacy Scale, and the Innovative Behavior Scale. Latent profile analysis (LPA) was employed to classify students into distinct digital literacy profiles, while logistic regression and one-way ANOVA were used to explore factors influencing profile membership and the relationship between digital literacy and innovative behavior.

**Results:**

Three latent profiles were identified: a “Low Digital Literacy” group (34.1%), a “Moderate Digital Literacy” group (15.9%), and a “High Digital Literacy” group (50.0%). Significant differences were observed across profiles in relation to gender, age, academic year, and frequency of artificial intelligence (AI) use in the past 6 months. Importantly, students with higher digital literacy consistently exhibited stronger innovative behavior (*p* < 0.05), underscoring the role of digital competence in fostering creativity and adaptability.

**Conclusion:**

Digital literacy among undergraduate nursing students is heterogeneous and shaped by demographic and experiential factors. Targeted educational interventions tailored to distinct literacy profiles are needed to bridge gaps, promote equity, and strengthen innovation. By integrating AI and advanced digital tools into nursing curricula, educators can enhance students’ competencies and better prepare them to thrive in an increasingly digital and intelligent healthcare landscape.

## 1 Introduction

As global digital transformation accelerates, information technology is profoundly altering societal models and ushering humanity into an era of ubiquitous connectivity and advanced human-machine intelligence (1–4). Globally, major countries and organizations increasingly view digital literacy as a strategic component of workforce development (5–8). In healthcare, digital technologies continue to transform service delivery models, intensifying the need for digitally competent nurses (9, 10). Nurses must integrate professional knowledge with digital competencies such as information management, medical software operation, and remote communication (11). These skills enhance decision-making, problem-solving, and research capacity, and support evidence-based practice (12). Studies in Switzerland and Ethiopia highlight gaps in training and low levels of digital literacy among health professionals, underscoring the urgency of reform. Yet current educational models often fail to meet practical needs, with curricula insufficiently integrating digital competencies (13–16). Medical educators must deeply consider how to effectively integrate digital literacy into conventional medical curricula and clinical practice.

## 2 Background

Digital literacy, a concept first introduced by Gilster (17), refers to the ability to access, evaluate, and apply digital information and is increasingly recognized as a foundation for evidence-based practice, clinical decision-making, and collaboration in technology-driven environments (17, 18). The European Union’s Digital Competence Framework further outlines five domains—information and data literacy, communication and cooperation, digital content creation, safety, and problem-solving (19). Understanding these dimensions and the diverse characteristics of nursing students is essential for integrating digital literacy into nursing education in China.

Innovation represents another essential capability in nursing education and practice. Defined as the generation, evaluation, and implementation of new ideas, innovative behavior encompasses activities from idea creation to securing support and enacting solutions (20–22). International guidelines, such as those in Norway, position innovation, health technology, and digital competence as core elements of nursing curricula (23). Recent studies consistently demonstrate reciprocal relationship between digital literacy and innovative capacity, particularly in AI-enhanced learning environments, where students with stronger digital skills demonstrate greater creativity, problem-solving, and confidence (24–26). This synergy enables nursing students to explore emerging career paths in medical technology, research, and management, reinforcing professional competence and adaptability in rapidly evolving health systems.

Self-determination theory provides a useful framework for understanding how digital literacy drives innovation. By enhancing perceived competence, autonomy, and relatedness, digital literacy strengthens intrinsic motivation and promotes innovative thinking (27, 28). Nursing students with higher digital literacy feel more confident and are more likely to embrace emerging technologies and adapt effectively to evolving clinical environments (29), thereby contributing to improved patient outcomes, efficient workflows, and continuous innovation (30–32). Demographic and experiential factors such as gender, education level, grade, artificial intelligence interest, usage frequency, and prior training further influence digital competence, highlighting the need for tailored educational strategies (33, 34).

Despite growing research on digital literacy in nursing education, most studies adopt variable-centered approaches, such as regression or structural equation modelling, that assume population homogeneity and overlook meaningful subgroup differences (35, 36). Evidence concerning Chinese undergraduate nursing students remains especially limited, despite their rapid exposure to digital technologies and evolving educational expectations. A person-centered method such as latent profile analysis (LPA) can identify unobserved subgroups with distinct digital literacy patterns, offering more actionable insights for precision teaching (37).

Therefore, this study applies LPA to classify digital literacy profiles among Chinese undergraduate nursing students, identify demographic and experiential determinants, and examine associations with innovative behavior. Specifically, the study aims to address three questions: (1) What distinct latent profiles of digital literacy exist among undergraduate nursing students? (2) How do student characteristics vary across these profiles? (3) Do levels of innovative behavior differ among the identified profiles?

## 3 Methods

### 3.1 Study subjects

This cross-sectional study recruited 450 undergraduate nursing students from one provincial key university and three provincial general universities in Anhui Province, China, between February and May 2025, using convenience sampling. These universities differ in location, academic focus, faculty strength, and student demographics, resulting in variation in access to learning resources and emphasis on professional skills. Inclusion criteria were: (1) full-time undergraduate nursing students currently enrolled; (2) voluntary participation; and (3) no history of mental illness or psychotropic drug use. Exclusion criteria were: (1) students on leave or withdrawn during the survey period; and (2) incomplete questionnaire responses.

To ensure classification accuracy, a minimum of 50 participants per category was required for LPA. Assuming 3–5 potential categories, the minimum sample size was set at 250 (38). Following the principle that multivariate analysis requires 10–20 times the number of observed variables, and given 21 variables (15 demographic and six scale dimensions), the estimated sample size ranged from 210 to 420 (39). Allowing for 10.0% attrition, the final sample size was determined to be between 231 and 462 participants.

### 3.2 Research tools

#### 3.2.1 General information questionnaire

A 13-item questionnaire was developed based on a literature review and study objectives, covering gender, age, academic year, home address, only-child status, parental education, household income, reasons for choosing nursing, leadership experience, and other relevant factors. Given the growing impact of AI on digital literacy (40), items also assessed interest in AI, prior AI education, and frequency of AI use in the past 6 months.

#### 3.2.2 Undergraduate students’ digital literacy scale

Digital literacy was measured using the Undergraduate Digital Literacy Scale developed by Liu (41), based on the European Union’s 2017 framework. The scale comprises five dimensions—privacy and security, information, communication and collaboration, problem solving, and digital content creation—across 22 items. Responses were rated on a five-point Likert scale (1 = strongly disagree to 5 = strongly agree), with higher scores indicating greater digital literacy. Cronbach’s *α* in this study was 0.944.

#### 3.2.3 The innovative behavior scale

The Innovative Behavior Scale was originally developed by Scott and Bruce (42) and revised by Liu and Shi (43), resulting in a standardized employee scale comprising one dimension with five items. The scale conceptualizes innovative behavior as a multi-phase process that includes generating new ideas, soliciting support for innovation, and implementing innovation plans. It uses a five-point Likert rating system ranging from “strongly disagree” to “strongly agree,” with scores from 1 to 5; higher scores indicate greater innovation. For this study, the items were adapted to the undergraduate nursing context. For example, “I often have creative ideas or suggestions at work” was modified to “I often have creative ideas or suggestions in my study and daily life.” Similarly, “I promote my new ideas to leaders or colleagues in order to gain their support and recognition” was revised to “I promote my new ideas to classmates, teachers, or family and friends in order to gain their support and recognition.” In this study, the Cronbach’s *α* coefficient for the scale was 0.897.

### 3.3 Data collection

Data were collected using an online survey platform^1^ between January 15 and November 8, 2024. An informed consent form accompanied each questionnaire, and only those who consented proceeded to complete the survey. The survey required 10–15 min; responses submitted in less than 90 s were excluded as invalid. To maintain data integrity, each IP address was restricted to a single submission. All items were mandatory to prevent missing data, and questionnaires showing obvious response patterns were excluded from analysis.

### 3.4 Statistical methods

LPA of undergraduate nursing students’ digital literacy was conducted using Mplus 8.3. Models were estimated with increasing numbers of profiles until the best-fitting solution was identified. Model fit was evaluated using log-likelihood value (LL), Akaike Information Criterion (AIC), Bayesian Information Criterion (BIC), adjusted BIC (aBIC), entropy, the Lo–Mendell–Rubin likelihood ratio test (LMRT), and the bootstrapped likelihood ratio test (BLRT). Lower AIC, BIC, and aBIC values indicated better fit (44); entropy values closer to 1 reflected higher classification accuracy (45); and significant LMRT and BLRT results (*p* < 0.05) suggested better model fit with k versus k–1 classes (46).

According to Curran et al. (47), data are approximately normal when skewness ranges from 2 to 2 and kurtosis from −7 to 7. In this study, skewness ranged from −0.729 to 0.170 and kurtosis from −0.443 to 0.837, indicating a near-normal distribution. SPSS 27.0 was used for further analysis. Continuous variables were reported as mean ± SD and compared using the independent t-test or one-way ANOVA; categorical variables were described by frequency and percentage, with group comparisons made using the chi-square test. Multivariate logistic regression identified factors influencing digital literacy categories. A one-way ANOVA was used to examine the association between categories and innovative behavior. Statistical significance was set at *p* < 0.05.

## 4 Results

### 4.1 Common method deviation test

Because this study relied on self-reported data, the potential for common method bias was assessed using the Harman single-factor test (48). Four common factors with eigenvalues greater than one were extracted. The variance explained by the first factor was 46.3%, which was below the critical threshold of 50.0% (49). These findings indicate that common method bias was not a major concern.

### 4.2 Participant characteristics

A total of 450 questionnaires were distributed, and after excluding 42 invalid responses, 408 valid questionnaires were retained, yielding an effective response rate of 90.7%. The majority of participants were female (76.7%), under 20 years of age (73.3%), and in their first or second year of study (93.6%). Additional demographic and study-related characteristics are presented in Table 1.

**Table 1:** Demographic and study-related features by latent profile membership (*n* = 408).

Variable	Total (*n* = 408)	“Low Digital Literacy” group (*n* = 139)	“Moderate Digital Literacy” group (*n* = 65)	“High Digital Literacy” group (*n* = 204)	*χ* ^2^	*p*
Gender						
Male	95 (23.3%)	43 (30.9%)	6 (9.2%)	46 (22.5%)	11.804	0.003
Female	313 (76.7%)	96 (69.1%)	59 (90.8%)	158 (77.5%)		
Age (years)						
≥20	109 (26.7%)	40 (28.8%)	27 (41.5%)	42 (20.6%)	11.508	0.003
<20	299 (73.3%)	99 (71.2%)	38 (58.5%)	162 (79.4%)		
Academic year						
Freshman	247 (60.5%)	80 (57.6%)	32 (49.2%)	135 (66.2%)	15.488	0.004
Sophomore	135 (33.1%)	43 (30.9%)	30 (46.2%)	62 (30.4%)		
Junior/Senior	26 (6.4%)	16 (11.5%)	3 (4.6%)	7 (3.4%)		
Home address						
City	91 (22.3%)	31(22.3%)	16 (24.6%)	44 (21.6%)	3.737	0.443
County seat	154 (37.7%)	57 (41.0%)	18 (27.7%)	79 (38.7%)		
Rural area	163 (40.0%)	51 (36.7%)	31 (47.7%)	81 (39.7%)		
Only-child status						
No	324 (79.4%)	111 (79.9%)	55 (84.6%)	158 (77.5%)	1.573	0.455
Yes	84 (20.6%)	28 (20.1%)	10 (15.4%)	46 (22.5%)		
Education level of father						
Junior high school and below	250 (61.3%)	76 (54.7%)	45 (69.2%)	129 (63.2%)	9.196	0.163
High school or technical secondary school	108 (26.5%)	38 (27.3%)	14 (21.5%)	56 (27.5%)		
Junior college	23 (5.6%)	13 (9.4%)	3 (4.6%)	7 (3.4%)		
University and above	27 (6.6%)	12 (8.6%)	3 (4.6%)	12 (5.9%)		
Education level of mother						
Junior high school and below	292 (71.6%)	95 (68.3%)	50 (76.9%)	147 (72.1%)	2.719	0.852
High school or technical secondary school	86 (21.1%)	31 (22.3%)	13 (20.0%)	42 (20.6%)		
Junior college	17 (4.2%)	7 (5.0%)	1 (1.5%)	9 (4.4%)		
University and above	13 (3.2%)	6 (4.4%)	1 (1.5%)	6 (2.9%)		
Reasons for choosing a nursing program						
Parents’ opinion	130 (31.9%)	48 (34.5%)	16 (24.6%)	66 (32.4%)	10.946	0.090
Major transfer	77 (18.9%)	34 (24.5%)	15 (23.1%)	28 (13.7%)		
Recommendation from others	124 (30.4%)	33 (23.7%)	23 (23.7%)	68 (33.3%)		
Personal interest	77 (18.9%)	24 (17.3%)	11 (17.3%)	42 (20.6%)		
Experience of student leader						
No	126 (30.9%)	41 (29.5%)	22 (33.8%)	63 (30.9%)	0.393	0.839
Yes	282 (69.1%)	98 (70.5%)	43 (66.2%)	141 (69.1%)		
Participation in social practice during the school year						
No	92 (22.5%)	31 (22.3%)	13 (20.0%)	48 (23.5%)	0.359	0.836
Yes	316 (77.5%)	108 (77.7%)	52 (80.0%)	156 (76.5%)		
Grade point average in required courses						
Pass (≥60 points)	110 (27.0%)	45 (32.4%)	16 (24.6%)	49 (24.0%)	8.632	0.195
Fair (≥70 points)	158 (38.7%)	51 (36.7%)	32 (49.2%)	75 (36.8%)		
Good (≥80 points)	122 (29.9%)	36 (25.9%)	16 (24.6%)	70 (34.3%)		
Excellent (≥90 points)	18 (4.4%)	7 (5.0%)	1 (1.5%)	10 (4.9%)		
Whether they have received any education in artificial intelligence						
No	267 (65.4%)	95 (68.3%)	47 (72.3%)	125 (61.3%)	3.440	0.179
Yes	141 (34.6%)	44 (31.7%)	18 (27.7%)	79 (38.7%)		
The frequency of using artificial intelligence in the past 6 months						
Rarely used	45 (11.0%)	25 (18.0%)	4 (6.2%)	16 (7.8%)	10.563	0.032
Moderately used	206 (50.5%)	49 (35.3%)	26 (40.0%)	82 (40.2%)		
Frequently used	157 (38.5%)	65 (46.8%)	35 (53.8%)	106 (52.0%)		
Whether is interested in artificial intelligence						
No	110 (27.0%)	45(32.4%)	17 (26.2%)	48 (23.5%)	3.310	0.191
Yes	298 (73.0%)	94 (67.6%)	48 (73.8%)	156 (76.5%)		
Daily online time						
<3 h	27 (6.6%)	9 (6.5%)	0 (0.0%)	18 (8.8%)	6.766	0.149
3-6 h	238 (58.3%)	78 (56.1%)	42 (64.6%)	118 (57.8%)		
>6 h	143 (35.0%)	52 (37.4%)	23 (35.4%)	68 (33.3%)		

### 4.3 Latent profiles of digital literacy among undergraduate nursing students

Latent profile models ranging from one to five classes were fitted using the 22 items of the digital literacy scale as observable indicators (Table 2). As the number of profiles increased, the entropy values consistently exceeded 0.900, and the BLRT *p*-values were all less than 0.001, while the AIC, BIC, and aBIC values decreased steadily. However, when the number of profiles increased to four, the LMRT p-value exceeded 0.05, indicating no significant improvement in model fit compared to the three-profile model. In the three-profile model, the reductions in AIC, BIC, and aBIC leveled off, and these indices were lower than those for the one- and two-profile models. Additionally, the two-profile model failed to capture intermediate states or subgroup diversity, whereas the three-profile model provided stronger support for differentiated instruction and educational decision-making, allowing educators to better understand student differences and adjust teaching strategies dynamically (50). Considering both statistical fit and practical relevance, the three-profile model was selected as optimal. Average membership probabilities for the three classes were 0.964, 0.934, and 0.942, all greater than 0.800, indicating high classification reliability (Table 3).

**Table 2:** The fitting results of the latent profile model for digital literacy in undergraduate nursing student (*n* = 408).

Model	LL	AIC	BIC	aBIC	Entropy	BLRT	LMRT	Minimum Class Size(Proportion)
						*p*		
1 Profile	−9147.073	18382.145	18558.641	18419.022	–	–	–	–
2 Profile	−7016.733	14167.466	14436.221	14223.619	0.977	<0.0001	0.0012	34.56 (141)
3 Profile	−6658.490	13496.981	13857.995	13572.410	0.972	<0.0001	0.0408	15.93 (65)
4 Profile	−6457.451	13140.902	13594.176	13235.608	0.975	<0.0001	0.1955	10.29 (42)
5 Profile	−6252.394	12776.787	13322.320	12890.769	0.980	<0.0001	0.1799	0.25 (1)

LL, Log Likelihood Value; AIC, Akaike Information Criterion; BIC, Bayesian Information Criterion; LMRT, Lo–Mendell–Rubin Likelihood Ratio Test; BLRT, Bootstrapped Likelihood Ratio Test.

**Table 3:** The probability of attribution of three potential profiles (*n* = 408).

Class	C1	C2	C3
C1	0.987	0.002	0.011
C2	0.004	0.978	0.018
C3	0.004	0.006	0.987

### 4.4 Designation of latent profiles of digital literacy among undergraduate nursing students

**Figure 1:**
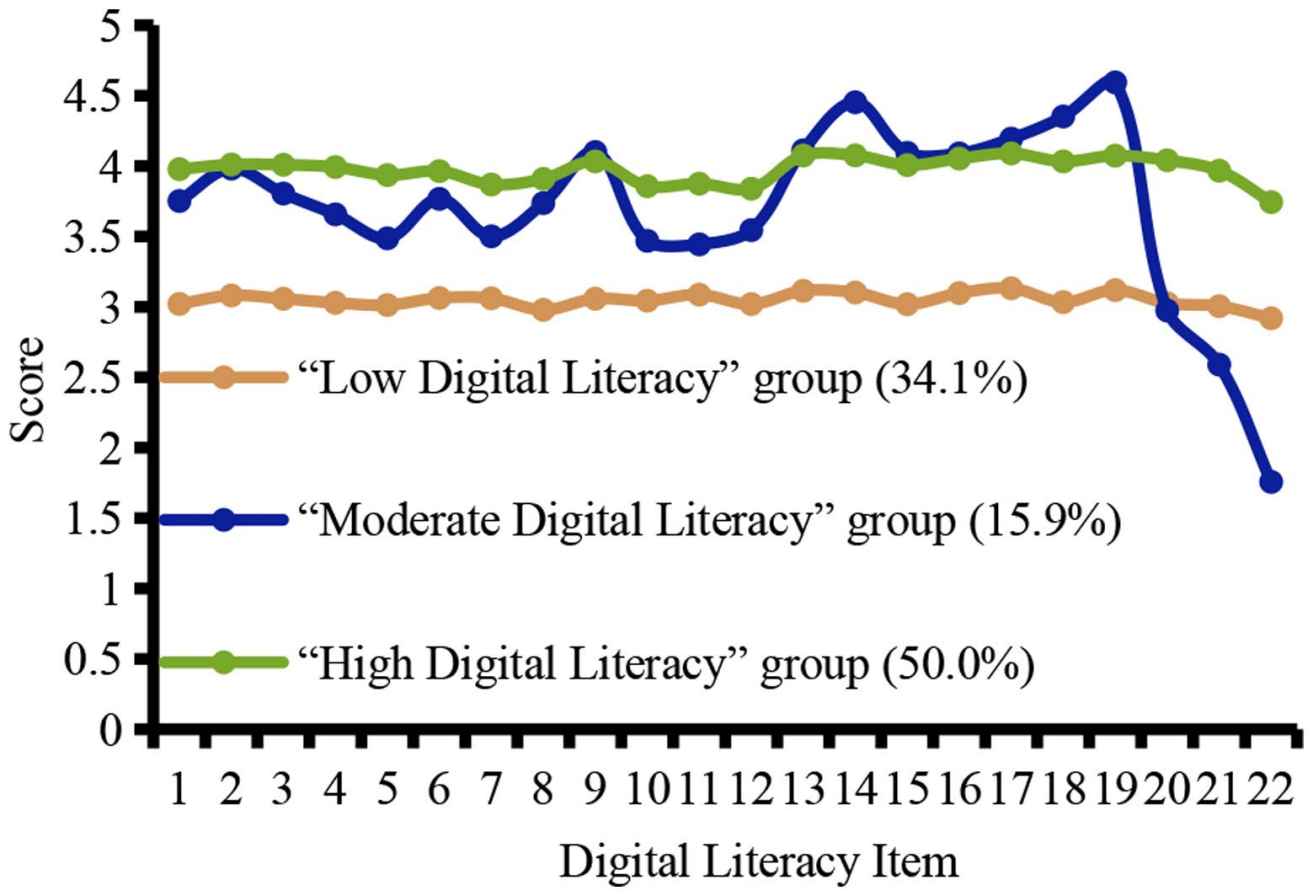
Potential profile category characteristics of digital literacy among undergraduate nursing students.

The distribution of scores for the 22 items across the five dimensions of the digital literacy scale for the three latent profiles of undergraduate nursing students is shown in Figure 1. Profile labels were assigned based on the characteristic item scores of each group. Profile 1 displayed the lowest overall level of digital literacy, with a mean score of 3.05 ± 0.22, and was designated the “Low Digital Literacy” group, comprising 139 participants (34.1%). Profile 2 showed considerable variation across specific dimensions, but its overall digital literacy level was higher than that of Profile 1 and lower than that of Profile 3, with a mean score of 3.71 ± 0.21. This group was therefore designated the “Moderate Digital Literacy” group, comprising 65 participants (15.9%). Profile 3 showed the highest overall level of digital literacy, with a mean score of 3.98 ± 0.18, and was designated the “High Digital Literacy” group, comprising 204 participants (50.0%).

### 4.5 Univariate analysis of latent profiles in digital literacy among undergraduate nursing students

This study examined the influence of demographic variables, including gender, age, academic year, home address, only-child status, and parental educational attainment, on the latent profile characteristics of digital literacy among undergraduate nursing students. The three latent profiles were treated as dependent variables, while the demographic characteristics served as independent variables. The analysis revealed significant differences (*p* < 0.05) across profiles with respect to gender, age, academic year, and frequency of AI use in the past 6 months. Detailed results are presented in Table 1.

### 4.6 Multivariate analysis of latent profiles in digital literacy among undergraduate nursing students

Multinomial logistic regression was conducted with the three latent profiles of digital literacy as dependent variables. To control for potential confounders and strengthen the explanatory power of the model, variables that were statistically significant in the univariate analyses (*p* < 0.05) were included as independent variables (51). The “High Digital Literacy” group was set as the reference category. Variable assignments are shown in Table 4, and regression outcomes are presented in Table 5. The analysis revealed that freshman (OR = 0.306, *p* = 0.022) and sophomore students (OR = 0.322, *p* = 0.028) were more likely to be categorized in the “High Digital Literacy” group. Students who reported infrequent use of AI in the past 6 months (OR = 2.581, *p* = 0.011) were more likely to fall into the “Low Digital Literacy” group. In addition, students under 20 years of age (OR = 0.368, *p* = 0.003) and male students (OR = 0.291, *p* = 0.009) had a higher likelihood of being classified in the “High Digital Literacy” group.

**Table 4:** Variable assignment status.

Variable	Assignment
Age (years)	≥20 = 0,<20 = 1
Gender	Male = 0, Female = 1
Academic year	Freshman = 0, Sophomore = 1, Junior/Senior = 2
The frequency of using artificial intelligence in the past 6 months	Rarely used = 0, Moderately used = 1, Frequently used = 2

**Table 5:** Logistic regression analysis of factors associated with potential profiles of latent digital literacy profiles in undergraduate nursing students (*n* = 408).

Variable	*β*	SE	Wald *χ*^2^	*p*	OR	95%CI
“Low Digital Literacy” group vs. “High Digital Literacy” group*						
Age (years)						
<20	−0.175	0.294	0.352	0.553	0.840	[0.472,1.495]
≥20*	–	–	–	–	–	–
Gender						
Male	0.401	0.256	2.454	0.117	1.494	[0.904,2.469]
Female*	–	–	–	–	–	–
Academic year						
Freshman	−1.184	0.517	5.251	0.022	0.306	[0.111,0.843]
Sophomore	−1.134	0.515	4.858	0.028	0.322	[0.117,0.882]
Junior/Senior*	–	–	–	–	–	–
The frequency of using artificial intelligence in the past 6 months						
Rarely used	0.948	0.374	6.433	0.011	2.581	[1.240,5.368]
Moderately used	0.008	0.245	<0.001	0.973	1.008	[0.623,1.631]
Frequently used*	–	–	–	–	–	–
“Moderate Digital Literacy” group vs. “High Digital Literacy” group*						
Age (years)						
<20	−0.999	0.339	8.658	0.003	0.368	[0.189,0.716]
≥20*	–	–	–	–	–	–
Gender						
Male	−1.235	0.472	6.839	0.009	0.291	[0.115,0.734]
Female*	–	–	–	–	–	–
Academic year						
Freshman	0.050	0.762	0.004	0.948	1.051	[0.236,4.676]
Sophomore	0.547	0.749	0.533	0.465	1.728	[0.398,7.508]
Junior/Senior*	–	–	–	–	–	–
The frequency of using artificial intelligence in the past 6 months						
Rarely used	−0.183	0.615	0.089	0.766	0.833	[0.249,2.779]
Moderately used	0.193	0.312	0.382	0.536	1.213	[0.658,2.233]
Frequently used*	–	–	–	–	–	–

*, Reference group; β, standardized coefficient.

### 4.7 Impact of different latent digital literacy profiles on innovative behavior among undergraduate nursing students

This study examined the relationship between the three latent profiles of digital literacy, treated as independent variables, and innovative behavior, designated as the dependent variable, among undergraduate nursing students. A one-way ANOVA was conducted, and the results indicated significant differences in mean innovative behavior scores across the profiles (*F* = 95.589, *p* < 0.001). Post-hoc pairwise comparisons showed that all differences among the profiles were statistically significant (*p* < 0.001). Students with higher levels of digital literacy consistently demonstrated greater innovative behavior. Detailed results are presented in Table 6.

**Table 6:** One-way ANOVA of innovative behavior among nursing students across different digital literacy profile categories.

Item	Innovative behavior
C1	15.55 ± 1.31
C2	17.54 ± 2.96
C3	18.85 ± 2.34
*F*	95.589**
Tamhane’s T2 Test	C3 > C1**
	C3 > C2**
	C2 > C1**

C1:“Low Digital Literacy” group; C2:“Moderate Digital Literacy” group; C3:“High Digital Literacy” group; **p < 0.001.

## 5 Discussion

### 5.1 Heterogeneity of digital literacy among undergraduate nursing students

The analysis indicated that students in the “Low Digital Literacy” group (34.1% of the total sample) often struggle with information retrieval, resource integration, and effective use of digital technologies. These deficits reflect broader socioeconomic disparities, uneven regional development, and the rapid pace of digital transformation in healthcare education (52). Such gaps reinforce the persistence of a digital divide that risks marginalizing certain groups of nursing students. Addressing this divide requires tailored educational strategies that promote equity and ensure all students can engage effectively with digital technologies. This aligns with the Digital Competence Framework (DigComp), which emphasizes inclusivity and progression from basic digital skills to advanced competencies (53). Nursing educators should therefore design interventions that gradually build foundational skills, ensuring that students with limited exposure are not left behind.

Students in the “Moderate Digital Literacy” group demonstrate awareness of digital risks and security issues but show limited independent problem-solving capacity. Despite making up only 15.9% of the sample, their profile highlights the importance of strengthening competencies in digital information security literacy and adaptive learning for nurses (54). In the context of nursing education, this group represents a transitional stage between basic and advanced digital competence. According to DigComp, information security and problem-solving are critical dimensions of digital literacy, and deficiencies in these areas may hinder students’ ability to manage complex technological tasks (53). Educational strategies should therefore focus on bridging these gaps, enhancing digital competency, and preparing students to manage complex tasks in digital nursing. From the perspective of the Technology Acceptance Model (TAM) (55), this group may perceive digital tools as useful but not sufficiently easy to use, which limits their confidence in independent application.

Students in the “High Digital Literacy” group (50.0% of the total sample) exhibit strong proficiency in acquiring, evaluating, and applying digital information. For this group, educators should move beyond basic training and provide advanced opportunities in areas such as informatics, AI-assisted decision-making, and healthcare big-data applications and electronic health record systems (56, 57). Advancing the digital literacy of this group could significantly enhance nursing practice and contribute to the transformation of healthcare in the modern era of AI-driven systems. Within the Diffusion of Innovation Theory, these students can be considered “early adopters,” positioned to lead the integration of digital technologies into nursing education and practice (58).

### 5.2 Factors influencing the latent profiles of digital literacy

Several demographic and experiential factors influence the likelihood of students belonging to different digital literacy profiles. Younger students, especially those in the early stages of their academic careers, are more likely to belong to the “High Digital Literacy” group. This reflects their exposure to digital transformation initiatives in higher education and supports the idea that early integration of digital training fosters stronger competencies (59, 60). In contrast, upper-year students often prioritize clinical practice (internships) and career preparation, which may limit their engagement with emerging technologies (34). Peer mentoring models, where digitally proficient younger students support their senior counterparts, could help bridge this gap (61). Such approaches resonate with the Diffusion of Innovation Theory, which emphasizes the role of early adopters in facilitating the spread of new practices (58). Younger students, acting as “innovators” or “early adopters,” can accelerate the diffusion of digital literacy across cohorts (62).

Gender differences may also play a role in digital literacy. The study revealed that males were more predisposed than females to be categorized in the “High Digital Literacy” group. Historical patterns of digitalization positioned internet and computer use as male-dominated pursuits, which constrained female participation and limited familiarity with these technologies (63, 64). More recent studies continue to highlight gender disparities in digital utilization and access to resources, with insufficient education identified as a critical factor restricting women’s and girls’ ability to capitalize on opportunities created by digital transformation (65). The TAM offers a useful theoretical lens for interpreting these disparities. TAM emphasizes perceived ease of use and perceived usefulness as key drivers of technology adoption (66). Evidence suggests that men are generally more self-confident with new technologies, more aware of their career relevance, and more willing to invest time in learning them (67, 68). These attitudes and behaviors contribute to men’s greater proficiency in accessing digital learning resources and adopting new technologies, thereby promoting higher levels of digital literacy. Taken together, these findings underscore the need for equity-focused interventions in nursing education. Educators should prioritize closing gender gaps in digital literacy by providing targeted support for female students, ensuring that underrepresented populations can fully benefit from digital transformation and thrive in inclusive digital environments.

Our findings confirm that the frequency of technology use is a critical predictor of digital competence (69). Students who reported infrequent use of AI were more likely to demonstrate lower levels of digital literacy, underscoring the importance of regular engagement with emerging technologies in shaping digital skills. Constructivist learning theory offers a valuable framework for interpreting this relationship. The theory emphasizes that learners build knowledge through active exploration, practice, and reflection in authentic contexts (70). Frequent hands-on engagement with AI not only strengthens technical expertise but also promotes deeper understanding through experiential Learning (71). As experience accumulates, learners’ competence in applying AI technologies increases, enhancing both technical skills and confidence in digital environments (72).

In contrast, students with limited exposure to AI lack opportunities to develop these competencies. Restricted practice hinders their effective use of information technology in complex tasks and clinical settings, ultimately constraining digital literacy. Given the accelerating integration of AI into healthcare systems, this gap represents a significant challenge for nursing education. AI has therefore emerged as a key driver of digital literacy development. Nursing educators should actively integrate AI applications into curricula, creating opportunities for students to explore, apply, and reflect on digital technologies in authentic learning environments. Such strategies will not only strengthen technical proficiency but also foster adaptive problem-solving and innovation, ensuring that future nurses are equipped to thrive in digitally transformed healthcare systems (73).

### 5.3 The association between digital literacy profiles and innovative behaviors in undergraduate nursing students

The study also highlights the strong association between digital literacy and innovative behavior. Students with higher digital literacy consistently demonstrated greater capacity for innovation, underscoring the transformative potential of digital competence in fostering creativity and problem-solving. This finding aligns with research showing that digital literacy enhances creative thinking by improving information assessment skills and inspiring innovative approaches to problem-solving (73). The relationship between digital literacy and innovation can be understood through the cognitive process framework developed by Reinhold et al. (74). This framework contends that digital technology serves not only as a medium for knowledge transmission but also as a crucial tool for deep information processing and the development of learners’ knowledge structures. In nursing education, digital tools such as virtual simulation platforms and intelligent decision support systems allow students to imitate clinical scenarios, examine patient cases, and engage in reflective practice. These tools promote ongoing enhancement of innovative behaviors and critical thinking skills in authentic clinical environments (75–78).

## 6 Advantages and limitations

The primary strength of this study lies in its use of latent profile analysis to identify subtypes of digital literacy, combined with rigorous statistical methods that enhance the reliability of the findings. The multicenter cross-sectional design further strengthens the representativeness of the sample and supports the generalizability of the results. Importantly, the study clarifies factors influencing digital literacy subtypes and explores their relationship with innovative behavior, providing valuable insights for nursing education in the context of digital transformation.

Nevertheless, several limitations must be acknowledged. The use of convenience sampling may have introduced selection bias, limiting the representativeness of the findings. The cross-sectional design also restricts causal inference, as associations between digital literacy and innovative behavior cannot be interpreted as causal relationships. Future research should adopt longitudinal designs to track changes in digital literacy and innovation across students’ academic progression, providing deeper insights into developmental trajectories and potential bidirectional interactions. Another limitation is the cultural specificity of the sample, which consisted exclusively of Chinese nursing students. The findings may therefore reflect unique features of China’s educational system, curriculum structure, and cultural context. To enhance generalizability, future studies should expand sample sizes and include participants from diverse regions, institutions, and educational backgrounds. Cross-cultural and cross-regional comparative studies would provide a more comprehensive understanding of digital literacy and innovation in nursing education, strengthening the international relevance of the findings.

## 7 Conclusion

This study identified three latent profiles of digital literacy among undergraduate nursing students: low, moderate, and high. These profiles highlight the heterogeneity of digital competence within nursing education and provide a framework for understanding how digital literacy relates to innovative behavior. The findings underscore the importance of recognizing diversity in digital skills and tailoring educational strategies accordingly. Future nursing educators must remain responsive to advances in digital technology and the evolving demands of the profession. By designing targeted training and intervention strategies that address the specific needs of each digital literacy profile, educators can foster both equity and excellence. Such approaches will not only strengthen students’ digital competencies but also enhance their capacity for innovation, thereby preparing them to thrive in an increasingly digital and AI-driven healthcare landscape.

## Data Availability

The raw data supporting the conclusions of this article will be made available by the authors, without undue reservation.

## Footnotes

1
https://www.wjx.cn/
